# Supplementary material for: Autoreactive IgG levels and Fc receptor γ subunit upregulation drive mechanical allodynia after nerve constriction or crush injury
Source: bioRxiv. 2025 Mar 24:2025.03.22.644748. Preprint. [Version 1] doi: 10.1101/2025.03.22.644748 (PMC11974762; doi:10.1101/2025.03.22.644748)
Supplement: Supplement 1 [file media-1.pdf]

Supplementary Materials:

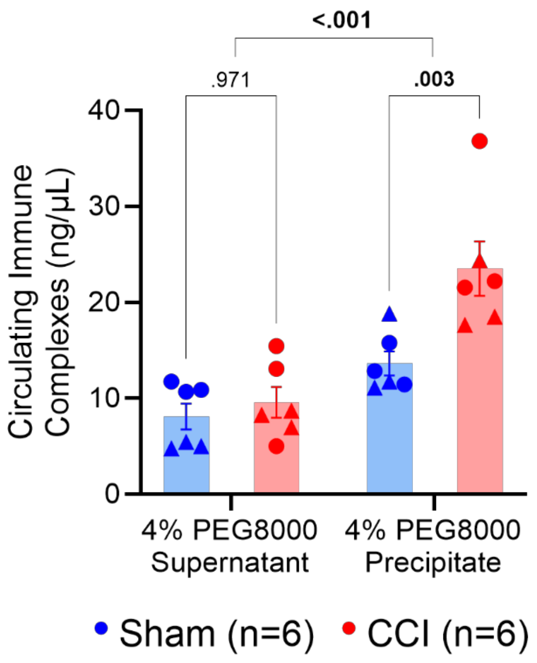

Figure S1. CCI increases circulating IgG ICs.

Quantification of circulating IgG ICs protein levels isolated from serum following 4% PEG8000 precipitation via ELISA. ELISA analyzed by two-way ANOVA and Dunnett's post hoc test; *p* values shown for comparisons CCI vs Sham and Precipitate vs Supernatant. *n* = 6/group (3 males and 3 females). For all data points, circles (●) represent male and triangles (▲) represent female mice.

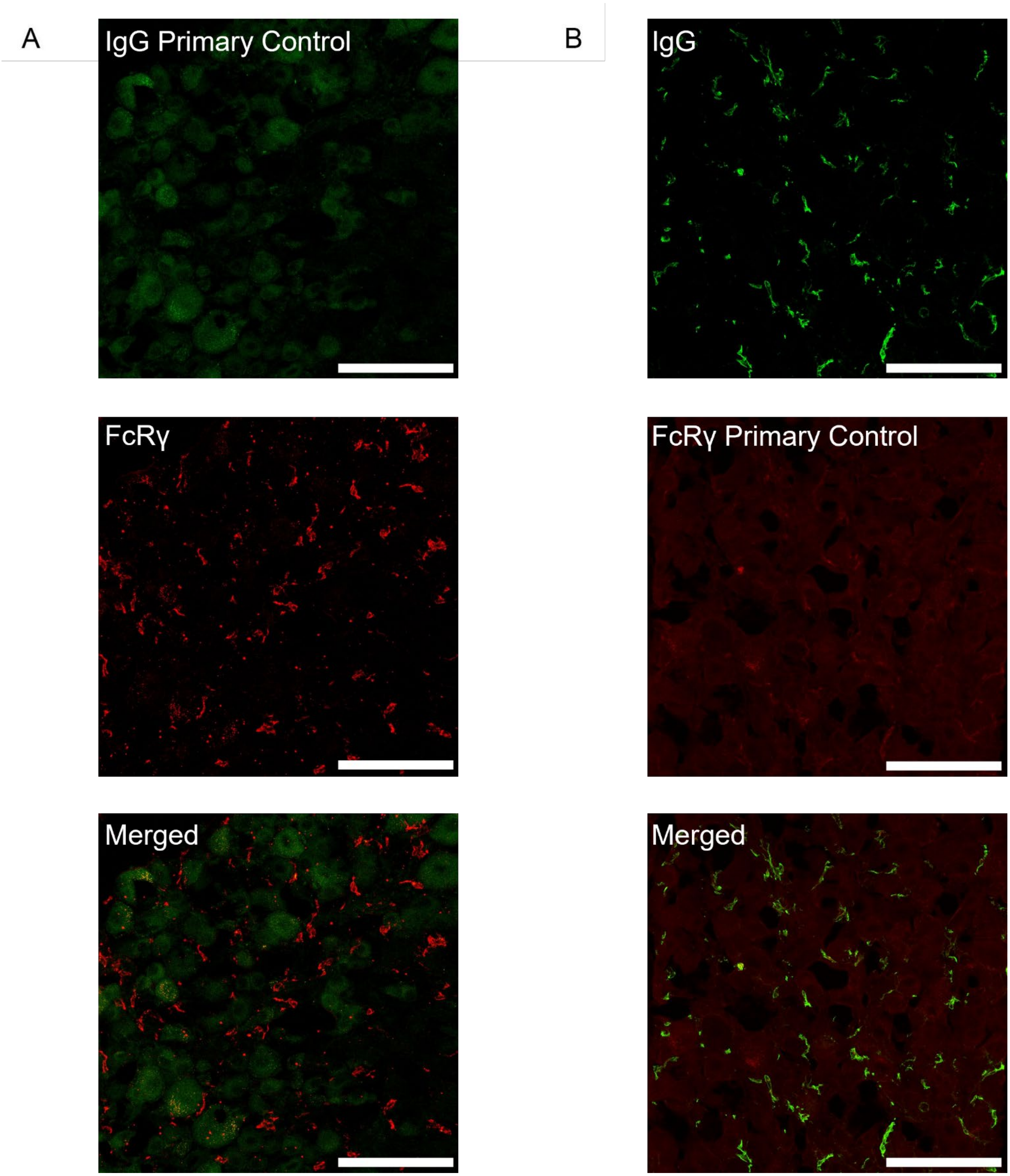

Figure S2. IgG and FcRγ antibody controls.

(**A**) IgG and (**B**) FcRγ antibody specificity was confirmed using primary antibody controls in DRG WT mice. Representative 40x fluorescent images (male, day 14 after surgery). Scale bars indicate 100μm.

Table S1. List of antibodies and primers.

Antibody / Primer	Vendor	Catalog#	Dilution/Conc
Goat anti-mouse IgG, $F(ab')_2$ fragment specific	Jackson ImmunoResearch	115-005-072	1:100
Rabbit anti-Map2	Abcam	ab32454	1:250
Mouse anti-FcRγ	MBL Life sciences	M191-3	1:200
Rabbit anti-NK.1	Invitrogen	PA132229	1:100
Donkey anti-goat IgG (H+L), Alexa Fluor 488	Invitrogen	A-11055	1:500
Donkey anti-rabbit IgG (H+L), Alexa Fluor 488	Invitrogen	A-21206	1:500
Donkey anti-mouse IgG (H+L), Alexa Fluor 594	Invitrogen	A-21203	1:500
Donkey anti-rabbit IgG (H+L), Alexa Fluor 594	Invitrogen	A-21207	1:500
DAPI (hydrochloride)	Sigma Aldrich	D9542	1:5000
Fcer1g Forward Mouse: GTATTGTCCTTACCCTACTCTAC	Sigma Aldrich	KSPQ12012G	500nM
Fcer1g Reverse Mouse: TGCTTCAGAGTCTCATATGTC	Sigma Aldrich	KSPQ12012G	500nM
Gapdh Forward Mouse: GTTTGTGATGGGTGTGAACC	Invitrogen	A15609	500nM
Gapdh Reverse Mouse: TCTTCTGAGTGGCAGTGATG	Invitrogen	A15609	500nM
